# Time‐weighted blood pressure with cardiovascular risk among patients with or without diabetes

**DOI:** 10.1002/clc.24213

**Published:** 2024-01-16

**Authors:** Zhixin Jiang, Fang Shao, Jingwen Hu, Qinyuan Zhuang, Pestheruwe Liyanaralalage Rosemarrie Krisunika Cooray, Kangyu Chen, Zhenqiang Wu, Tao Chen, Chao Li

**Affiliations:** ^1^ Department of Cardiology The First Affiliated Hospital of Nanjing Medical University Nanjing China; ^2^ Department of Biostatistics Nanjing Medical University School of Public Health Nanjing Jiangsu China; ^3^ School of International Education Nanjing Medical University Nanjing Jiangsu China; ^4^ Department of Cardiology Division of Life Sciences and Medicine, The First Affiliated Hospital of USTC University of Science and Technology of China Hefei China; ^5^ Department of Geriatric Medicine The University of Auckland Auckland New Zealand; ^6^ Centre for Health Economics University of York York UK; ^7^ Department of Clinical Sciences Liverpool School of Tropical Medicine Liverpool UK; ^8^ Department of Epidemiology and Health Statistics School of Public Health, Xi'an Jiaotong University Health Science Centre Xi'an China

**Keywords:** blood pressure, cardiovascular events, diabetes, hypertension

## Abstract

**Background:**

Usual measures of blood pressure (BP) do not account for both the magnitude and duration of exposure to elevated BP over time. We aimed to demonstrate the effect of a novel time‐weighted BP on cardiovascular outcomes using a post hoc analysis of two published randomized trials.

**Hypothesis:**

Time‐weighted blood pressure is associated with cardiovascular risk among patients with or without diabetes.

**Methods:**

The limited‐access ACCORD and SPRINT data sets were used for the current study. Time‐weighted BP is obtained by dividing cumulative BP by the total follow‐up time. Time‐weighted BP burden above a threshold is also determined after deriving the time‐weighted BP by re‐zeroing the interpolated pressure values at two different hypertension thresholds (>140/90 and >130/80 mmHg).

**Results:**

Eighteen thousand five hundred forty‐one patients from the two clinical trials were enrolled in this study. A J‐curve relation was observed between time‐weighted BP and major cardiovascular events (MACE). The systolic blood pressure (SBP) burden independently predicted MACE across the two trials at different thresholds (ACCORD: SBP > 130 mmHg, HR = 1.05 [1.03−1.06]; SBP > 140 mmHg, HR = 1.06 [1.04−1.08]; SPRINT: SBP > 130 mmHg, HR = 1.04 [1.03−1.05]; SBP > 140 mmHg, HR = 1.05 [1.04−1.07]). Consistent results were found for diastolic blood pressure (DBP) burden (ACCORD: DBP > 80 mmHg, HR = 1.10 [1.06−1.15]; DBP > 90 mmHg, HR = 1.20 [1.11−1.30]. SPRINT: DBP > 80 mmHg, HR = 1.06 [1.02−1.09]; DBP > 90 mmHg, HR = 1.12 [1.06−1.18]). Significant associations were also observed for stroke, myocardial infarction, cardiovascular death, and all‐cause mortality.

**Conclusion:**

Both time‐weighted SBP and DBP independently influenced the risk of adverse cardiovascular events among patients with and without diabetes, regardless of the definition of hypertension (130/80 or <140/90 mmHg).

## Keypoints

- A novel approach was introduced to assess the time‐weighted systolic blood pressure (SBP) and diastolic blood pressure (DBP) as predictors of major adverse cardiovascular events (MACE).
- Both time‐weighted SBP and DBP independently influenced the risk of MACE in patients with and without diabetes, regardless of how hypertension was defined (<130/80 or <140/90 mmHg).
- This study highlights the significance of consistently maintaining adequate control of both SBP and DBP throughout an individual's lifespan to prevent cardiovascular events.

## Graphical abstract

This study employed data sourced from the ACCORD and SPRINT clinical trials to unveil the correlation between time‐weighted blood pressure and the incidence of significant adverse cardiovascular events in individuals with diabetes and those without diabetes. Parts of the figure were drawn by using pictures from Servier Medical Art. Servier Medical Art by Servier is licensed under a Creative Commons Attribution 3.0 Unported License (https://creativecommons.org/licenses/by/3.0/).

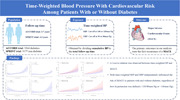

**Abbreviations**
ACCORD: action to control cardiovascular risk in diabetes
BMI: body mass index
BP: blood pressure
Cis: confidence intervals
DBP: diastolic blood pressure
eGFR: estimated glomerular filtration rate
HDL‐C: high‐density lipoprotein cholesterol
HF: heart failure
HR: hazard ratio
LDL‐C: low‐density lipoprotein cholesterol
MACE: major cardiovascular events
MI: myocardial infarction
SBP: systolic blood pressure
SPRINT: systolic blood pressure intervention trial

## 1 INTRODUCTION

Control of blood pressure (BP) has been a central focus of international guidelines for the prevention of myocardial infarction (MI), stroke, heart failure (HF), and other cardiovascular morbidities.^1^, ^2^, ^3^, ^4^ Because the management of hypertension has traditionally centered around BP measurements taken at a single time point, it fails to recognize BP as a continuous measure that fluctuates over time. Dynamic BP measurements may play an important role in cardiovascular risk prediction.^5^, ^6^, ^7^ Compared to a single BP assessment, the inclusion of cumulative BP provided incremental prognostic value and improved risk reclassification for cardiovascular events.^8^, ^9^, ^10^ The cumulative BP integrated both the magnitude and duration in units of mm Hg × time. However, this measure cannot differentiate the same cumulative BP in the form of a very high BP level with a short follow‐up and a high BP level with a long follow‐up.

In acute HF patients, 24‐hour time‐weighted lactate had a greater predictive value than the other static and dynamic indices of lactate homeostasis.^11^ Similarly, time‐weighted BP is obtained by dividing cumulative BP by the total follow‐up time, which takes into account the amount of time spent at every single BP in relation to the total period of time observed.^9^, ^12^, ^13^ As the predictive value of time‐weighted BP remains elusive, we aim to explore the effect of time‐weighted systolic blood pressure (SBP) and diastolic blood pressure (DBP) on the risk of major cardiovascular events (MACE) in patients with or without diabetes on the basis of a post hoc analysis of two published randomized trials, ACCORD^14^, ^15^ (Action to Control Cardiovascular Risk in Diabetes) and the SPRINT^16^, ^17^ (Systolic Blood Pressure Intervention Trial).

Our objective is to gain a deeper understanding of the impact of time‐weighted SBP and DBP on the risk of MACE and to compare these effects in patients with and without diabetes by conducting this study. This research will advance the knowledge of the role dynamic BP measurements play in the development and prognosis of cardiovascular diseases, which will help to improve precise guidance for personalized hypertension management. The findings of this study may contribute to enhancing BP management strategies, which would ultimately lower the prevalence of cardiovascular diseases and their relevant adverse effects.

## 2 METHODS

### 2.1 Trial design and oversight

The limited‐access ACCORD and SPRINT data sets obtained from the NIH upon approval were used for the current post hoc analysis. The details of two randomized, controlled trials have been reported previously.^14^, ^15^, ^16^, ^17^ In general, the ACCORD trial was a double two‐by‐two factorial, parallel treatment trial in 10 251 patients with type 2 diabetes. All participants in the ACCORD were randomly assigned to one of two glycemic treatment arms, with the standard arm targeting HbA1c 7.0%−7.9% and the intensive targeting HbA1c < 6.0% (ACCORD glycemia trial). Half of the participants (5518) of the ACCORD participants were also allotted to a lipid intervention consisting of randomization to fenofibrate versus placebo (ACCORD lipid trial). The other half (4733) of participants were either intensive (SBP ≤ 120 mmHg) or standard (SBP ≤ 140 mmHg) BP treatment (ACCORD‐BP trial). The mean duration of follow‐up was 3.7 years in the ACCORD trial. The SPRINT study was a randomized, open‐label, multicenter trial. Nine thousand three hundred sixty‐one high‐risk patients were assigned to either intensive or standard BP treatment group similar to those used in the ACCORD‐BP trial, with a median follow‐up of 3.26 years. In contrast with the ACCORD trial, the participants in the SPRINT study excluded those with diabetes.

Additionally, both trials were approved by the institutional review board or ethics committee at each study site and all participants provided written informed consent. This post hoc analysis was waived for ethical approval by the ethical committee of the Liverpool School of Tropical Medicine (No:20‐077). In our current analysis, we excluded 887 patients from the ACCORD trial and 184 patients from SPRINT trial because of missing data or lost follow‐up.

### 2.2 BP measurement

In both trials, BP was measured by the automated device (Model 907; Omron Healthcare) while the participant was seated. In the SPRINT trial, BP measurements were taken monthly in the first 3 months and every 3 months thereafter. In the ACCORD trial, patients allocated to the intensive BP treatment group were seen every month for 4 months and every 2 months thereafter. In the standard BP treatment group, visits were scheduled at 1 and 4 months and then every 4 months thereafter. Participants not enrolled in the ACCORD‐BP trial were seen every 4 months starting from 4 months after the intervention.

### 2.3 Time‐weighted BP and BP burden

The calculation of time‐weighted SBP and DBP was based on all available BP measurements before any incidence of MACE or from baseline to the end of both trials. It was obtained by dividing cumulative BP by the total follow‐up time. After determining the time‐weighted BP, the time‐weighted BP burden above a threshold is determined by re‐zeroing the interpolated pressure values at the threshold (130/140 mmHg for SBP, 80/90 mmHg for DBP) measured from the 6‐month visit until their last visit or the visit before an event (Appendix Figure S1). The 6‐month visit time point was chosen because of the stability of BP values after this visit.

### 2.4 Outcomes

The primary outcomes for the ACCORD and SPRINT trials, adopted in our analysis, were the first occurrence of a MACE. For the ACCORD trial, the definition of primary outcome was nonfatal MI, nonfatal stroke, or cardiovascular death. For the SPRINT trial, the MACE was defined as MI, acute coronary syndrome not resulting in MI, stroke, acute decompensated HF, or cardiovascular death. The secondary outcomes in our analysis included stroke, MI, HF, cardiovascular death, and all‐cause mortality.

### 2.5 Statistical analysis

We used the Poisson regression model to estimate the relationship between time‐weighted BP and incidence rate of MACE in the ACCORD and SPRINT studies after adjusting for the variables of age, sex, race, treatment group, history of clinical CVD, history of dyslipidemia, history of hypertensive, current smoking, current drinking, body mass index (BMI), baseline SBP, estimated glomerular filtration rate (eGFR), glucose, HDL‐C, and LDL‐C. In the Poisson regression model, time‐weighted BP was categorized into <110 mmHg, [110−115] mmHg, [115−120] mmHg, [120−125] mmHg, [125−130] mmHg, [130−135] mmHg, [135−140] mmHg, [140−145] mmHg, [145−140] mmHg, ≥150 mmHg for SBP, and <50 mmHg, [50−55] mmHg, [55−60] mmHg, [60−65] mmHg, [65−70] mmHg, [70−75] mmHg, [75−80] mmHg, [80−85] mmHg, 85−90] mmHg, ≥90 mmHg for DBP. The predicted number of MACE events was estimated in each subgroup, and the incidence rate was calculated by the predicted number of MACE events divided by survival time accordingly.

We used the Cox model to explore the time‐weighted BP with the outcomes of interest by two models (Model 1: adjusting age, sex, race, current smoking, current drinking, BMI; Model 2: Model 1 + history of clinical CVD, history of dyslipidemia, history of hypertensive treatment, BMI, baseline SBP, eGFR, glucose, HDL‐C, LDL‐C. To further explore the relationship between time‐weighted BP burden and MACE in both trials, we employed spline analysis within the Cox model, which included SBP burden (≥130/140 mmHg) and DBP burden (≥80/90 mmHg) as natural cubic splines to account for a continuous nonlinear functional dependence. We specified 0 mmHg as the reference value for SBP and DBP burden. Spline knots were placed at the 5th, 50th, and 95th centiles of the overall distribution of BP burden.

We also performed a series of sensitivity analyses including repeated analysis for secondary outcomes and among different subgroups of participants (ACCORD‐BP trial participants, ACCORD participants with noncontrolled BP, Intensive BP‐controlled SPRINT participants, Standard BP‐controlled SPRINT participants) to calculate hazard ratios (HRs) and 95% confidence intervals (CIs). All analyses were performed using STATA version 15.0 (Stata Corporation).

## 3 RESULTS

A total of 18 541 participants from the two clinical trials were assessed in this cohort study. Among the participants, White individuals constituted the highest proportion (60.06%), followed by Black individuals (24.37%). Table 1 shows the baseline characteristics for the participants from the subgroups of BP burden (≥140/90 or ≥130/80 mmHg). In general, baseline characteristics in higher BP burden tend to be older age, also a higher percentage of black race, stroke, and HF history.

**Table 1:** Baseline characteristics of study population in the ACCORD and SPRINT trial.^a^

	ACCORD			SPRINT		
	Total	Mean blood pressure^b^	Mean blood pressure^b^	Total	Mean blood pressure^b^	Mean blood pressure^b^
		(≥140/90 mmHg)	(≥130/80 mmHg)		(≥140/90 mmHg)	(≥130/80 mmHg)
	9364	1452	4244	9177	1158	4830
Demographics						
Age (years)	62.66 ± 6.59	63.57 ± 6.86	62.99 ± 6.71	67.91 ± 9.40	68.61 ± 10.44	67.61 ± 9.67
Female	3644 (38.91)	589 (40.56)	1634 (38.50)	3251 (35.43)	467 (40.33)	1721 (35.63)
Race						
Black	1778 (18.99)	399 (27.48)	970 (22.86)	2734 (29.79)	431 (37.22)	1584 (32.80)
White	5826 (62.22)	767 (52.82)	2445 (57.60)	5310 (57.86)	609 (52.59)	2686 (55.50)
Hispanic	684 (7.30)	136 (9.37)	339 (7.99)	967 (10.54)	95 (8.20)	476 (9.86)
Others	1076 (11.49)	150 (10.33)	490 (11.55)	166 (1.81)	23 (1.99)	84 (1.74)
Medical history						
CHD	2660 (28.41)	397 (27.34)	1152 (27.14)	1274 (13.88)	145 (12.52)	609 (12.61)
Stroke^c^	549 (5.86)	116 (7.99)	270 (6.36)	‐	‐	‐
HF^c^	422 (4.51)	78 (5.37)	176 (4.15)	‐	‐	‐
Dyslipidemia	6536 (69.80)	963 (66.32)	2918 (68.76)	3986 (43.43)	449 (38.77)	2048 (42.40)
Current smoking	1282 (13.69)	206 (14.19)	582 (13.71)	1212 (13.22)	200 (17.29)	672 (13.93)
Current drinking	2253 (24.06)	314 (21.63)	981 (23.11)	3243 (35.47)	430 (37.33)	1696 (35.25)
Treatment						
Intensive BP treatment (%)	2135 (22.80)	90 (6.20)	304 (7.16)	4600 (50.13)	178 (15.37)	879 (18.20)
Intensive glycemic treatment^d^ (%)	4726 (50.47)	675 (46.49)	2071 (48.80)	‐	‐	‐
Intensive lipid treatment^d^ (%)	2525 (26.96)	442 (30.44)	1140 (26.86)	‐	‐	‐
Biometric and laboratory data						
BMI (kg/m)^2^	32.27 ± 5.39	32.21 ± 5.42	32.23 ± 5.33	29.81 ± 5.64	29.06 ± 5.64	29.70 ± 5.55
SBP (mm Hg)	136.16 ± 16.96	150.50 ± 16.64	143.52 ± 15.84	139.65 ± 15.58	148.25 ± 16.76	141.93 ± 15.71
DBP (mm Hg)	74.96 ± 10.57	78.45 ± 11.42	77.47 ± 10.76	78.13 ± 11.92	80.79 ± 14.29	79.41 ± 12.50
eGFR (mL/min/1.73 m)^2^	91.33 ± 27.21	88.75 ± 24.44	90.41 ± 25.39	71.76 ± 20.58	70.05 ± 22.45	71.57 ± 20.95
Glucose (mmol/L)	9.80 ± 3.13	10.03 ± 3.40	9.92 ± 3.29	5.53 ± 0.76	5.51 ± 0.79	5.53 ± 0.76
HDL‐C (mmol/L)	1.09 ± 0.30	1.08 ± 0.31	1.08 ± 0.30	1.37 ± 0.37	1.41 ± 0.40	1.37 ± 0.38
LDL‐C (mmol/L)	2.70 ± 0.87	2.79 ± 0.90	2.76 ± 0.87	2.91 ± 0.91	2.95 ± 0.91	2.94 ± 0.91

*Note*: Categorical variables are reported as percentages of the characteristic. Continuous variables are reported as mean ± SD.

Abbreviations: BMI, body mass index; BP, blood pressure; CHD, coronary heart disease; DBP, diastolic blood pressure; eGFR, estimated glomerular filtration rate; HDL‐C, high‐density lipoprotein cholesterol; HF, heart failure; LDL‐C, low‐density lipoprotein cholesterol; SBP, systolic blood pressure.

^a^ Missing value of time‐weighted blood pressure has been deleted from current analysis.

^b^ Time‐weighted blood pressure is used to estimate mean blood pressure.

^c^ The population with a past medical history of stroke or heart failure has been excluded from the SPRINT trial.

^d^ Only intensive or standard SBP treatments were conducted in the SPRINT trial.

**Figure 1:**
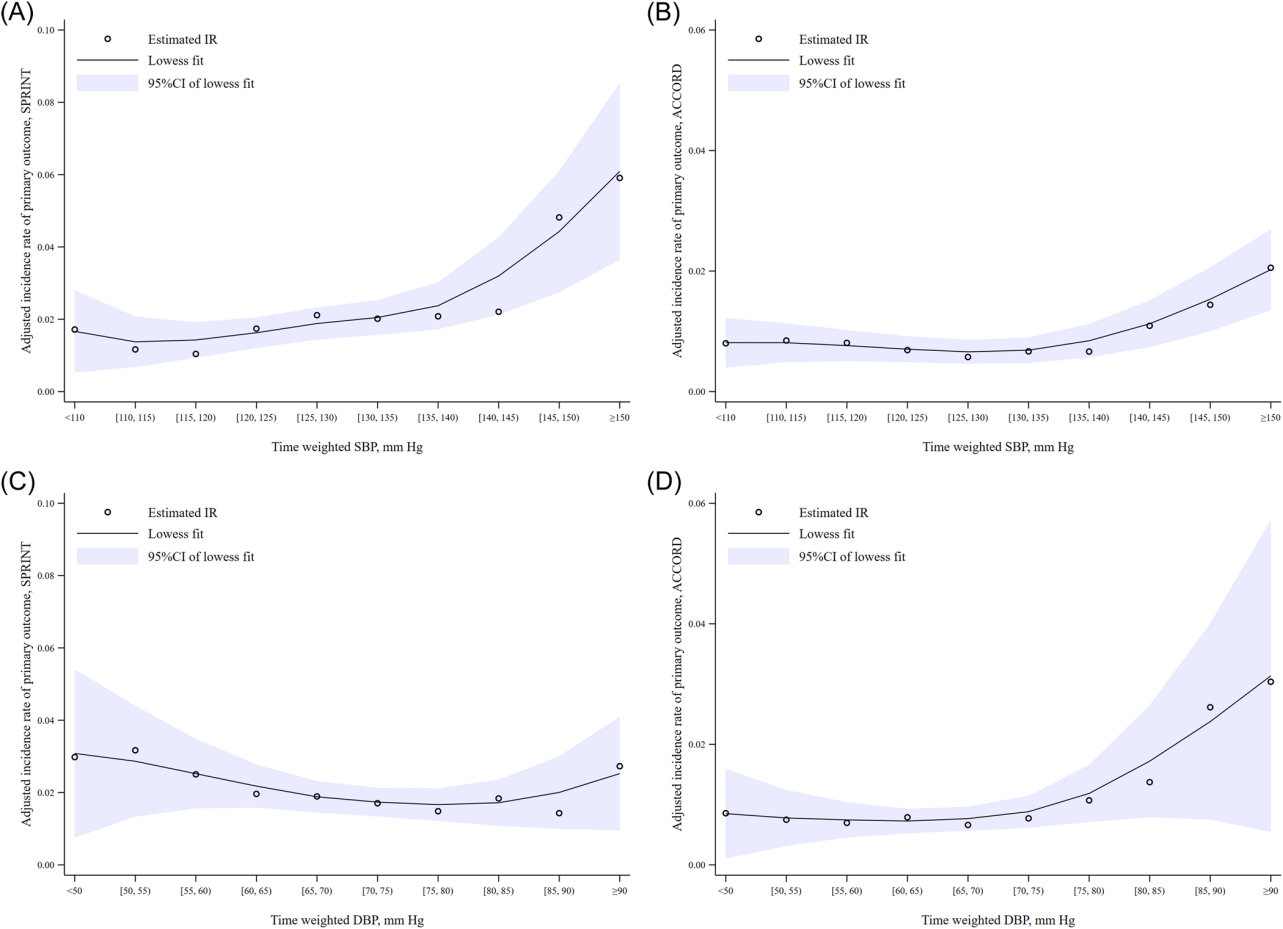
Adjusted relationships between time‐weighted BP (categorical variables) and incidence rate of primary outcome in the SPRINT and ACCORD trials. The relationships were estimated by using Poisson regression after adjusting the variables of age, sex, race, treatment group, history of clinical CVD, history of dyslipidemia, history of hypertensive treatment, current smoking, current drinking, BMI, baseline SBP, eGFR, glucose, HDL‐C, LDL‐C. The Lowess method (the shadow represents the upper and lower bounds of 95% CI) was used to connect the incidence rate of the primary outcome in each time‐weighted SBP or DBP subgroup. BMI, body mass index; BP, blood pressure; CVD, cardiovascular disease; DBP, diastolic blood pressure; eGFR, estimated glomerular filtration rate; HDL‐C, high‐density lipoprotein cholesterol; LDL‐C, low‐density lipoprotein cholesterol; SBP, systolic blood pressure.

In general, a J‐curve relation was seen between time‐weighted SBP or DBP and the composite outcome. In both the ACCORD and SPRINT cohorts, the incidence rate began to increase when SBP exceeded 130 mmHg and DBP exceeded 80 mmHg. Interestingly, in the SPRINT cohort, there was a significantly faster increase in the risk of cardiovascular events following SBP exceeding 130 mmHg compared to the ACCORD cohort. This could be attributed to the fact that individuals with diabetes are inherently more susceptible to cardiovascular events (Figure 1). Our results from the Cox model further demonstrated that the SBP burden (≥140 mmHg) significantly increased the incidence of MACE, stroke, MI, HF, CVD death, and all‐cause mortality in both ACCORD and SPRINT trials (Table 2). The DBP burden (≥90 mmHg) was also independently associated with the composite outcome, HF, CVD death, and all‐cause mortality in the two trials. What's more, the association between a high DBP burden (≥90 mmHg) and stroke was found to be statistically significant only in the ACCORD trial. Similarly, the statistically significant association between a high DBP burden (≥90 mmHg) and MI was observed only in the SPRINT trial (Table 3). When we set the threshold for BP burden at 130/80 mmHg, the results are consistent with the aforementioned findings. The sensitivity analysis results support the stability of the research findings (Appendix Tables S1 and S2). There was no statistically significant correlation that appeared to be found between the incidence rate of MACE and the last BP measured before MACE or baseline BP, with the exception of DBP (≥80 mmHg), and this was consistent across both cohorts (Appendix Table S3).

**Table 2:** Associations between SBP (≥130/140 mmHg) burden and cardiovascular events in the ACCORD and SPRINT trials.

	ACCORD		SPRINT	
	Model 1	Model 2	Model 1	Model 2
	HR (95% CI)	HR (95% CI)	HR (95% CI)	HR (95% CI)
Primary outcome				
SBP burden (≥130 mmHg)	1.04 (1.03−1.06)	1.05 (1.03−1.06)	1.05 (1.04−1.06)	1.04 (1.03−1.05)
SBP burden (≥140 mmHg)	1.05 (1.04−1.07)	1.06 (1.04−1.08)	1.06 (1.05−1.08)	1.05 (1.04−1.07)
Stroke				
SBP burden (≥130 mmHg)	1.09 (1.06−1.11)	1.08 (1.05−1.12)	1.08 (1.06−1.10)	1.08 (1.05−1.10)
SBP burden (≥140 mmHg)	1.10 (1.07−1.13)	1.09 (1.06−1.13)	1.09 (1.07−1.12)	1.08 (1.06−1.11)
MI				
SBP burden (≥130 mmHg)	1.03 (1.01−1.06)	1.06 (1.03−1.09)	1.05 (1.02−1.07)	1.04 (1.01−1.06)
SBP burden (≥140 mmHg)	1.05 (1.01−1.08)	1.07 (1.04−1.11)	1.06 (1.03−1.09)	1.05 (1.02−1.09)
HF				
SBP burden (≥130 mmHg)	1.04 (1.03−1.06)	1.05 (1.03−1.06)	1.06 (1.03−1.08)	1.05 (1.02−1.08)
SBP burden (≥140 mmHg)	1.06 (1.05−1.08)	1.06 (1.04−1.08)	1.08 (1.05−1.11)	1.07 (1.04−1.11)
CVD death				
SBP burden (≥130 mmHg)	1.03 (1.01−1.04)	1.03 (1.01−1.05)	1.07 (1.05−1.10)	1.06 (1.03−1.10)
SBP burden (≥140 mmHg)	1.04 (1.02−1.06)	1.04 (1.02−1.06)	1.09 (1.06−1.12)	1.08 (1.04−1.13)
All‐cause mortality				
SBP burden (≥130 mmHg)	1.02 (1.01−1.03)	1.03 (1.02−1.05)	1.04 (1.02−1.06)	1.03 (1.01−1.06)
SBP burden (≥140 mmHg)	1.04 (1.02−1.05)	1.04 (1.03−1.06)	1.06 (1.04−1.09)	1.05 (1.02−1.08)

*Note*: Model 1: adjusted for age, sex, race, current smoking, current drinking, BMI; Model 2: adjusted for age, sex, race, treatment group, history of clinical CVD, history of dyslipidemia, history of hypertension treatment, current smoking, current drinking, BMI, baseline SBP, eGFR, glucose, HDL‐C, LDL‐C.

Abbreviations: BMI, body mass index; CI, confidence interval; CVD, cardiovascular disease; DBP, diastolic blood pressure; eGFR, estimated glomerular filtration rate; HDL‐C, high‐density lipoprotein cholesterol; HF, heart failure; HR, hazard ratio; LDL‐C, low‐density lipoprotein cholesterol; MI, myocardial infarction; SBP, systolic blood pressure.

**Table 3:** Associations between DBP (≥80/90 mmHg) burden and cardiovascular events in the ACCORD and SPRINT trials.

	ACCORD		SPRINT	
	Model 1	Model 2	Model 1	Model 2
	HR (95% CI)	HR (95% CI)	HR (95% CI)	HR (95% CI)
Primary outcome				
DBP burden (≥80 mmHg)	1.10 (1.06−1.15)	1.10 (1.06−1.15)	1.05 (1.02−1.09)	1.06 (1.02−1.09)
DBP burden (≥90 mmHg)	1.21 (1.12−1.31)	1.20 (1.11−1.30)	1.13 (1.07−1.19)	1.12 (1.06−1.18)
Stroke				
DBP burden (≥80 mmHg)	1.24 (1.15−1.33)	1.21 (1.12−1.30)	1.10 (1.04−1.16)	1.03 (0.97−1.09)
DBP burden (≥90 mmHg)	1.39 (1.24−1.56)	1.33 (1.17−1.51)	1.09 (0.98−1.20)	1.07 (0.96−1.19)
MI				
DBP burden (≥80 mmHg)	0.93 (0.79−1.10)	0.96 (0.82−1.13)	1.03 (0.98−1.09)	1.06 (1.02−1.09)
DBP burden (≥90 mmHg)	0.84 (0.47−1.51)	0.89 (0.51−1.56)	1.13 (1.07−1.19)	1.12 (1.06−1.18)
HF				
DBP burden (≥80 mmHg)	1.09 (1.04−1.14)	1.08 (1.03−1.13)	1.07 (1.00−1.14)	1.06 (0.99−1.13)
DBP burden (≥90 mmHg)	1.20 (1.11−1.30)	1.16 (1.07−1.26)	1.18 (1.09−1.28)	1.16 (1.07−1.27)
CVD death				
DBP burden (≥80 mmHg)	1.08 (1.03−1.14)	1.09 (1.04−1.15)	1.13 (1.07−1.20)	1.13 (1.07−1.19)
DBP burden (≥90 mmHg)	1.17 (1.06−1.29)	1.15 (1.05−1.27)	1.25 (1.16−1.34)	1.23 (1.15−1.32)
All‐cause mortality				
DBP burden (≥80 mmHg)	1.08 (1.04−1.11)	1.08 (1.04−1.12)	1.07 (1.03−1.11)	1.06 (1.02−1.11)
DBP burden (≥90 mmHg)	1.14 (1.05−1.23)	1.13 (1.05−1.23)	1.15 (1.08−1.22)	1.13 (1.06−1.21)

*Note*: Model 1: adjusted for age, sex, race, current smoking, current drinking, BMI; Model 2: adjusted for age, sex, race, treatment group, history of clinical CVD, history of dyslipidemia, history of hypertensive treatment, current smoking, current drinking, BMI, baseline SBP, eGFR, glucose, HDL‐C, LDL‐C.

Abbreviations: BMI, body mass index; CI, confidence interval; CVD, cardiovascular disease; DBP, diastolic blood pressure; eGFR, estimated glomerular filtration rate; HDL‐C, high‐density lipoprotein cholesterol; HF, heart failure; HR, hazard ratio; LDL‐C, low‐density lipoprotein cholesterol; MI, myocardial infarction; SBP, systolic blood pressure.

**Figure 2:**
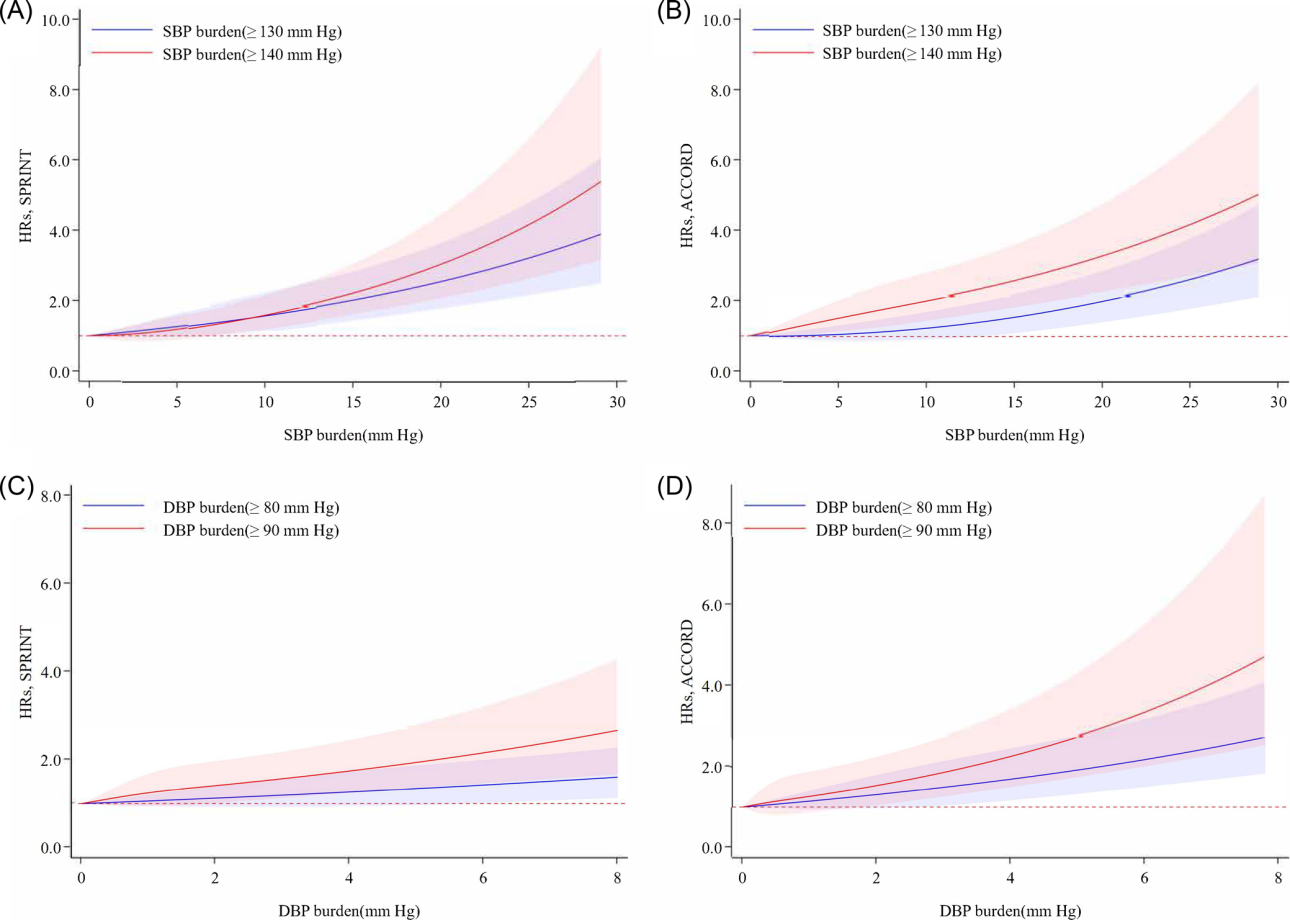
Spline analyses of SBP (≥130/140 mmHg) and DBP (≥80/90 mmHg) burden in the SPRINT and ACCORD trials. HRs for the combined primary outcome (shadow represent upper and lower bounds of 95% CI) is relative to 0 mmHg for SBP and DBP burden. Knots are placed at the 5th, 50th, and 95th centiles of the time‐weighted SBP and DBP. Multivariable model was adjusted for the variables of age, sex, race, treatment group, history of clinical CVD, history of dyslipidemia, history of hypertensive treatment, current smoking, current drinking, BMI, baseline SBP, eGFR, glucose, HDL‐C, LDL‐C. BMI, body mass index; CVD, cardiovascular disease; DBP, diastolic blood pressure; eGFR, estimated glomerular filtration rate; HDL‐C, high‐density lipoprotein cholesterol; LDL‐C, low‐density lipoprotein cholesterol; SBP, systolic blood pressure.

Additionally, while including BP burden as continuous, we observed a linear trend for SBP (≥130/140 mmHg) or DBP (≥80/90 mmHg) burden relative to 0 mmHg with MACE in both trials (Figure 2).

## 4 DISCUSSION

This study was to investigate the prognosis value of time‐weighted BP in a hypertensive population with or without diabetes. We found that the rate of MACE increased from the time‐weighted SBP ≥ 130 mmHg subgroups and time‐weighted DBP ≥ 80 mmHg subgroups. A J‐curve relation was observed between time‐weighted BP and MACE. These findings were similar between patients with or without diabetes, which could support tightened BP targets (<130/80 mmHg) for hypertensive patients.

Repeated assessments of BP, rather than a single baseline measure, would provide a more accurate representation of an individual's BP profile.^5^, ^18^, ^19^ Even in a well‐controlled hypertensive population, a normal distribution of BPs around the mean is a common phenomenon, while the MACE risk is determined by both the magnitude and the cumulative duration of exposure to high BP.^20^ Ambulatory BP measurement predicts fatal and nonfatal MI and stroke better than standard office measurement does.^5^ Day‐to‐day (office visit‐to‐visit) variability in BP has been proposed as an independent predictor of cardiovascular risk.^6^ In a recent study, cumulative SBP load was recognized as a superior predictor of MACE compared with mean SBP, SBP time at target, and SBP SD among patients with type 2 diabetes.^18^ In our study, both time‐weighted SBP and DBP were found to be efficient for cardiovascular risk prediction. This novel index describes not only the magnitude but also the duration and trend over the time of BP monitoring.

Many randomized control trials have proven the advantages received by type 2 diabetes patients by the reduction of BP.^21^, ^22^, ^23^ However, optimal BP levels continue to be debated in this special population. The landmark clinical trial ACCORD showed a similar cardiovascular risk between target SBP of <120 mmHg and target SBP of <140 mmHg in patients with diabetes.^14^ Furthermore, the 2017 ADA Position Statement on Diabetes and Hypertension does not promote a uniform BP target (<130/80 mmHg) and instead risk stratifies to avoid overtreatment in type 2 diabetes patients.^24^ Our results demonstrated a J‐curve relationship between time‐weighted BP and MACE with nadirs at SBP 130−135 mmHg or DBP 80−85 mmHg, which may support the recommendation of a target of BP < 130/80 mmHg.

Despite hypertension guidelines included in both SBP and DBP targets,^1^, ^2^, ^3^, ^25^ treatment for hypertension with measurement of only SBP has been argued on the basis of data from the Framingham Heart Study.^26^, ^27^ In a recent study using data from 1.3 million adults in a general outpatient population, both SBP and DBP independently influenced the risk of MACE, regardless of the definition of hypertension (≥140/90 or ≥130/80 mmHg).^28^ Our results also showed that time‐weighted SBP and DBP each independently influenced cardiovascular outcomes, and therefore DBP ought not to be ignored in patients with or without type 2 diabetes.

### 4.1 Limitations

The majority of the population in this study is from northern America. Additional external data is required to validate the results of our study. Potential randomized clinical trials guided by time‐weighted BP would be attested to improve unacceptable low BP control.

### 4.2 Future directions

There are numerous pathways for researching and exploring further in this field, although the current study yielded compelling evidence concerning the predictive value of time‐weighted BP for cardiovascular events. Initially, it would be vital to conduct longer‐term follow‐up studies with larger sample sizes to assess the sustained predictive capability of this measurement. Monitoring participants for a vast period would enable an ample assessment of the long‐term consequences of time‐weighted BP on cardiovascular outcomes.

## 5 CONCLUSION

Our study proposed a novel assessment of time‐weighted SBP and DBP for predicting the risk of MACE. Both time‐weighted SBP and DBP independently influenced the risk of MACE among patients with and without diabetes, despite the two distinct definitions of hypertension (defined as BP ≥ 140/90 or ≥130/80 mmHg). The findings of this study demonstrate the importance of adequate and sustained control of both SBP and DBP throughout life for prevention of cardiovascular events.

## CONFLICT OF INTEREST STATEMENT

The authors declare no conflict of interest.

## Supporting information

Supporting information.

Supporting information.

Supporting information.

Supporting information.

## Data Availability

Data sharing is not applicable to this article as no new data were created or analyzed in this study. The ACCORD and SPRINT clinical trial data used in this study can be obtained and granted permission for use through the appropriate application process.
