# Supplementary figures and images for: Time‐weighted blood pressure with cardiovascular risk among patients with or without diabetes
Source: Clin Cardiol. 2024 Jan 16;47(1):e24213. doi: 10.1002/clc.24213 (PMC10790318; doi:10.1002/clc.24213)

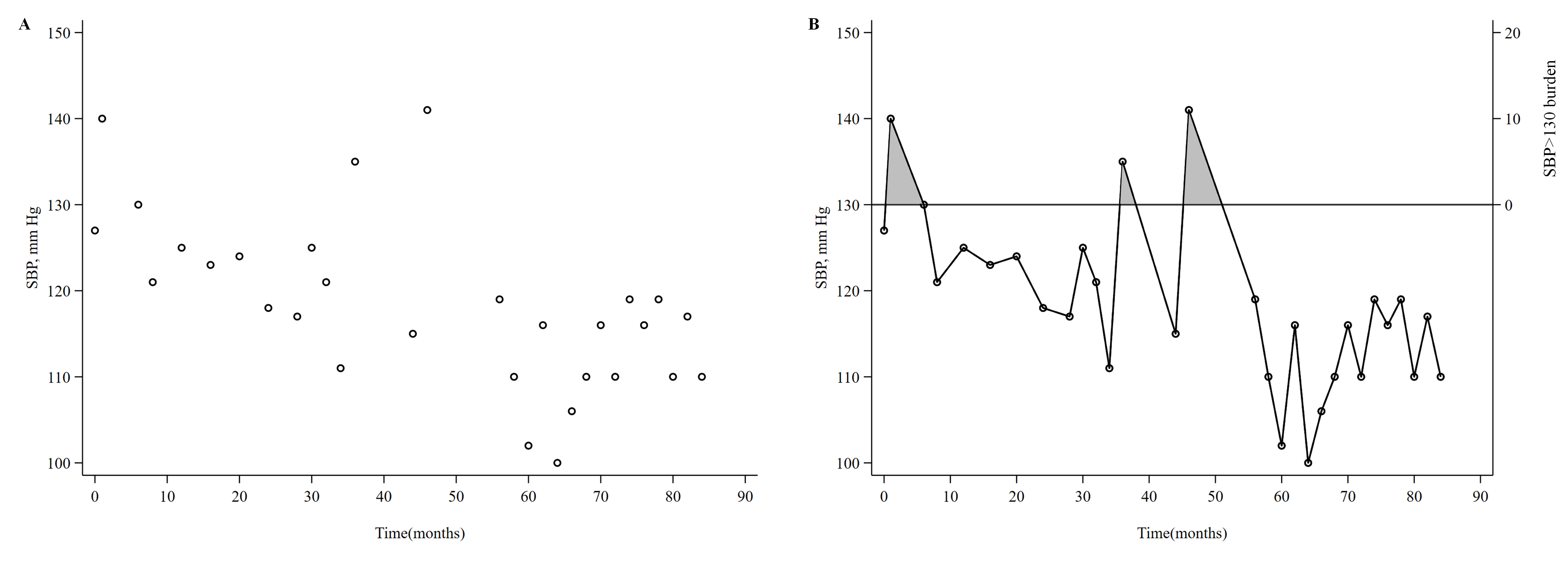

Supplement: Supplementary file 1 — Supporting information. [file CLC-47-e24213-s003.tif]
