# Supplementary material for: Time‐weighted blood pressure with cardiovascular risk among patients with or without diabetes
Source: Clin Cardiol. 2024 Jan 16;47(1):e24213. doi: 10.1002/clc.24213 (PMC10790318; doi:10.1002/clc.24213)
Supplement: Supplementary file 2 — Supporting information. [file CLC-47-e24213-s002.docx]

| **Appendix Table 1. Association between different SBP metrics and primary outcomes in the SPRINT and ACCORD trial** | | | | | | | |
| --- | --- | --- | --- | --- | --- | --- | --- |
|  | ACCORD | | |  | SPRINT | | |
|  | Total | BP Control trial | Non-BP Control trial |  | Total | Standard BP Control | Intensive BP Control |
|  | HR (95%CI) | HR (95%CI) | HR (95%CI) |  | HR (95%CI) | HR (95%CI) | HR (95%CI) |
| **Model 1** |  |  |  |  |  |  |  |
| SBP>130 mmHg |  |  |  |  |  |  |  |
| Time weighted | 1.04(1.03, 1.05) | 1.05(1.03, 1.07) | 1.04(1.02, 1.05) |  | 1.05(1.04, 1.06) | 1.07(1.06, 1.09) | 1.03(1.01, 1.05) |
| SBP>140 mmHg |  |  |  |  |  |  |  |
| Time weighted | 1.05(1.04, 1.07) | 1.07(1.04, 1.10) | 1.05(1.03, 1.07) |  | 1.07(1.05, 1.08) | 1.10(1.07, 1.13) | 1.05(1.03, 1.08) |
|  |  |  |  |  |  |  |  |
| **Model 2** |  |  |  |  |  |  |  |
| SBP>130 mmHg |  |  |  |  |  |  |  |
| Time weighted | 1.06(1.03, 1.08) | 1.04(1.03, 1.07) | 1.04(1.02, 1.05) |  | 1.05(1.03, 1.06) | 1.07(1.04, 1.09) | 1.03(1.01, 1.05) |
| SBP>140 mmHg |  |  |  |  |  |  |  |
| Time weighted | 1.06(1.04, 1.08) | 1.07(1.04, 1.11) | 1.06(1.04, 1.08) |  | 1.06(1.04, 1.08) | 1.08(1.05, 1.11) | 1.05(1.02, 1.08) |
|  |  |  |  |  |  |  |  |

Model 1: adjusted for age, sex, race, current smoking, current drinking, BMI; Model 2: adjusted for age, sex, race, history of clinical CVD, history of dyslipidemia, history of hypertensive, current smoking, current drinking, BMI, baseline SBP, eGFR, glucose, HDL-C, LDL-C.

BMI, Body mass index; CVD, Cardiovascular Disease; SBP, Systolic blood pressure; DBP, Diastolic blood pressure; eGFR, Estimated glomerular filtration rate; HDL-C, High-density lipoprotein cholesterol; LDL-C, Low-density lipoprotein cholesterol HF, Heart failure; MI: Myocardial infarction.
