# Supplementary material for: Time‐weighted blood pressure with cardiovascular risk among patients with or without diabetes
Source: Clin Cardiol. 2024 Jan 16;47(1):e24213. doi: 10.1002/clc.24213 (PMC10790318; doi:10.1002/clc.24213)
Supplement: Supplementary file 3 — Supporting information. [file CLC-47-e24213-s004.docx]

| **Appendix Table 2. Association between different DBP metrics and primary outcomes in the SPRINT and ACCORD trial** | | | | | | | |
| --- | --- | --- | --- | --- | --- | --- | --- |
|  | ACCORD | | |  | SPRINT | | |
|  | Total | BP Control trial | Non-BP Control trial |  | Total | Standard BP Control | Intensive BP Control |
|  | HR (95%CI) | HR (95%CI) | HR (95%CI) |  | HR (95%CI) | HR (95%CI) | HR (95%CI) |
| **Model 1** |  |  |  |  |  |  |  |
| DBP>80 mmHg |  |  |  |  |  |  |  |
| Time weighted | 1.10(1.06, 1.15) | 1.05(0.94, 1.16) | 1.11(1.06, 1.16) |  | 1.06(1.03, 1.10) | 1.08(1.03, 1.13) | 1.04(0.99, 1.08) |
| DBP>90 mmHg |  |  |  |  |  |  |  |
| Time weighted | 1.22(1.13, 1.32) | 1.22(0.96, 1.55) | 1.22(1.12, 1.32) |  | 1.13(1.07, 1.19) | 1.11(1.04, 1.19) | 1.16(1.05, 1.27) |
|  |  |  |  |  |  |  |  |
| **Model 2** |  |  |  |  |  |  |  |
| DBP>80 mmHg |  |  |  |  |  |  |  |
| Time weighted | 1.11(1.06, 1.15) | 1.05(0.94, 1.17) | 1.11(1.06, 1.16) |  | 1.07(1.03, 1.10) | 1.07(1.02, 1.12) | 1.05(1.01, 1.10) |
| DBP>90 mmHg |  |  |  |  |  |  |  |
| Time weighted | 1.20(1.11, 1.30) | 1.22(0.94, 1.59) | 1.19(1.09, 1.30) |  | 1.12(1.07, 1.18) | 1.09(1.01, 1.17) | 1.18(1.07, 1.29) |
|  |  |  |  |  |  |  |  |

Model 1: adjusted for age, sex, race, current smoking, current drinking, BMI; Model 2: adjusted for age, sex, race, history of clinical CVD, history of dyslipidemia, history of hypertensive treatment, current smoking, current drinking, BMI, baseline SBP, eGFR, glucose, HDL-C, LDL-C.

BMI, Body mass index; CVD, Cardiovascular Disease; SBP, Systolic blood pressure; DBP, Diastolic blood pressure; eGFR, Estimated glomerular filtration rate; HDL-C, High-density lipoprotein cholesterol; LDL-C, Low-density lipoprotein cholesterol.

*The HR and 95%CI cannot be estimated due to the few events(n=25) among participants with DBP>90 mm Hg.
