# Supplementary material for: Time‐weighted blood pressure with cardiovascular risk among patients with or without diabetes
Source: Clin Cardiol. 2024 Jan 16;47(1):e24213. doi: 10.1002/clc.24213 (PMC10790318; doi:10.1002/clc.24213)
Supplement: Supplementary file 4 — Supporting information. [file CLC-47-e24213-s001.docx]

| **Appendix Table 3. Association between different BP metrics and primary outcomes in the SPRINT and ACCORD trial** | | |
| --- | --- | --- |
|  | ACCORD  HR (95%CI) | SPRINT  HR (95%CI) |
| **SBP (mm Hg)** | | |
| **SBP>130** |  |  |
| Time-weighted | 1.06(1.03, 1.08) | 1.05(1.03, 1.06) |
| Baseline | 1.03(0.99, 1.07) | 1.02(0.99, 1.04) |
| Last | 1.03(0.99, 1.07) | 1.02(0.99, 1.04) |
| **SBP>140** |  |  |
| Time-weighted | 1.06(1.04, 1.08) | 1.06(1.04, 1.08) |
| Baseline | 1.01(0.99, 1.03) | 1.01(0.99, 1.03) |
| Last | 1.02(0.99, 1.05) | 1.02(0.98, 1.06) |
| **DBP (mm Hg)** | | |
| **DBP>80** |  |  |
| Time-weighted | 1.11(1.06, 1.15) | 1.07(1.03, 1.10) |
| Baseline | 1.02(0.99, 1.04) | 1.01(0.99, 1.03) |
| Last | 1.03(0.95, 1.11) | 1.03(0.97, 1.09) |
| **DBP>90** |  |  |
| Time-weighted | 1.20(1.11, 1.30) | 1.12(1.07, 1.18) |
| Baseline | 1.04(1.00, 1.10) | 1.03(1.00, 1.06) |
| Last | 1.05(0.92, 1.20) | 1.02(0.89, 1.17) |

Multivariable model was adjusted for the variables of age, sex, race, treatment group, history of clinical CVD, history of dyslipidemia, history of hypertensive, current smoking, current drinking, BMI, baseline SBP, eGFR, glucose, HDL-C,

LDL-C

HR: Hazard ratio; CI: Confidence Interval; BMI, Body mass index; CVD, Cardiovascular Disease; SBP, Systolic blood pressure; DBP, Diastolic blood pressure; eGFR, Estimated glomerular filtration rate; HLD-C, High-density lipoprotein cholesterol; LDL-C, Low-density lipoprotein cholesterol.
